# Risk and liability in the deployment of AI systems for surgery: a SAGES white paper

**DOI:** 10.1007/s00464-026-12881-8

**Published:** 2026-06-08

**Authors:** Daniel A. Hashimoto, Jayson S. Marwaha, Sharon A. Lee, Steven Schwaitzberg, Mindy N. Duffourc

**Affiliations:** 1 Department of Surgery, Perelman School of Medicine, University of Pennsylvania, 3400 Spruce Street, 4 Silverstein Pavilion, Philadelphia, PA 19104 USA; 2 Department of Computer and Information Science, School of Engineering and Applied Science, University of Pennsylvania, Philadelphia, PA USA; 3 Department of Surgery, University of Michigan, Ann Arbor, MI USA; 4 Department of Surgery, University of Buffalo, Buffalo, NY USA; 5 Faculty of Law, Maastricht University, Maastricht, Netherlands

**Keywords:** Artificial intelligence, Liability, Risk, Informed consent, Malpractice, Data privacy

## Abstract

**Background:**

Artificial intelligence (AI) is increasingly utilized in surgical care for decision support, operative planning, intraoperative guidance, and autonomous functions. While these systems can enhance efficiency and clinical performance, they also introduce risks related to technology, human factors, legal issues, and ethics. Current regulatory and legal frameworks are not fully equipped to address the challenges of AI-assisted surgery.

**Methods:**

This SAGES white paper synthesizes current regulatory, legal, ethical, and clinical considerations relevant to the deployment of AI systems in surgery. We review market safety regulation, data privacy law, informed consent, malpractice and liability principles, and propose a conceptual framework for understanding risk in surgical AI implementation.

**Results:**

Risks associated with surgical AI can be understood through a tripartite framework: risks inherent to the AI system, risks introduced by the clinician-user, and risks arising from institutional deployment. These risks may manifest clinically as diagnostic error, treatment error, compromised informed consent, erosion of patient trust, threats to therapeutic autonomy, and privacy violations. Although surgeons remain the ultimate clinical decision-makers, liability may also extend to developers for defective design or failure to warn, and to institutions for negligent implementation or oversight.

**Conclusion:**

The safe integration of AI into surgery requires more than technical performance alone. Robust governance, ongoing performance surveillance, incident response pathways, clinician credentialing, and specialty-society engagement are needed to reduce harm and clarify accountability.

Artificial intelligence (AI) and machine learning (ML)-based techniques are becoming increasingly prevalent in healthcare, where they have been shown to detect disease, improve the accuracy of diagnosis, identify patterns contributing to pathology, and offer intraoperative guidance. Article 3 of the European Union (EU) Artificial Intelligence Act defines an ‘AI system’ as “a machine-based system that is designed to operate with varying levels of autonomy and that may exhibit adaptiveness after deployment, and that, for explicit or implicit objectives, infers, from the input it receives, how to generate outputs such as predictions, content, recommendations, or decisions that can influence physical or virtual environments” [1]. AI systems can use techniques such as machine learning, defined as a system that has the capacity to learn based on training on a specific task by tracking performance measure(s) [2]. ML is characterized by continuous learning that adapts to new data to improve its overall performance, triggering the need for specific regulations that can accommodate its dynamic and iterative nature. It is increasingly crucial to consider the details surrounding surgical AI governance and liability, particularly when ML-based technologies are implemented within surgical environments, and AI-guided decisions may lead to significant and irreversible outcomes.

## Market safety regulation

Regulatory approval pathways of AI systems are intended to reduce risks associated with their clinical use. In response to the increased integration of AI/ML-based methods in healthcare, the United States (U.S.) Food and Drug Administration (FDA) and medical device regulators in the EU created frameworks to regulate the development and use of these technologies. These frameworks also build upon the International Medical Device Regulators Forum (IMDRF)’s efforts to harmonize regulatory requirements for medical products [3]. Broadly, the IMDRF defines software as a medical device (SaMD) as “software intended to be used for one or more medical purposes that perform these purposes without being part of a hardware medical device.” In 2019, the FDA adapted the SaMD framework to accommodate the highly iterative, autonomous, and adaptive nature of AI/ML-based SaMD while maintaining a reasonable level of assurance of device safety and effectiveness. The proposed regulatory framework for risk categorization is based on the (1) significance of the information provided by the SaMD to the healthcare decision to treat or diagnose, drive or inform clinical management, and (2) the state of the healthcare situation or condition specified as critical, serious, and non-serious. SaMD with higher levels of impact based on these criteria is subject to more rigorous marketing submissions for a device, risk and quality management, post-market surveillance, and more frequent risk assessments to determine if changes to the SaMD change its categorization and core functionality [4].

This updated framework provides a total product lifecycle (TPLC) regulatory approach that consists of:

1. Establishing clear expectations on quality systems and good ML practices, such as demonstrating a valid clinical association between the SaMD’s output and the targeted clinical condition and providing analytical and clinical validation;
2. Conducting premarket review for those SaMD that require premarket submission, including the development of a predetermined change control plan (PCCP) that includes the types of anticipated modifications based on a specified retraining and model updating strategy, which ensures the continued safety and effectiveness of a device without necessitating additional marketing submission for implementing each modification detailed in the PCCP;
3. Enforcing a risk management approach for resubmitting for approval pertaining to a software or algorithm change; and
4. Enabling increased transparency to the public using post-market real-world performance reporting and providing detailed documentation of any subsequent modifications [5].

In 2024, the EU supplemented sectoral regulation of AI/ML-based SaMD by adopting the Artificial Intelligence Act, which established a common framework for the use and supply of AI systems across the EU. The AI Act also uses risk-based regulatory classifications, stratifying by unacceptable, high, and minimal risk. Each risk level corresponds to different compliance requirements for testing, data training, risk management, and cybersecurity. It further enforces transparency requirements, such as disclosing the use of AI to generate content and providing publicly available summaries of the content used in training general-purpose AI systems [6]. These frameworks are intended to promote the development of safe and effective AI/ML-enabled devices without stymying their often rapid iterative performance enhancements.

## Data privacy regulation

Despite the vast potential of AI/ML-SaMD to enhance patient diagnostics, care, and therapeutics, it is important to ensure that individual data and privacy remain protected. The Health Insurance Portability and Accountability Act (HIPAA) Privacy Rule and General Data Protection Regulation (GDPR) are the two major Western privacy laws that protect individuals’ medical records and other individually identifiable health information or protected health information (PHI) [5, 7]. HIPAA requires appropriate safeguards to protect the privacy of PHI, setting limits and conditions on the uses and disclosures that may be made on such information without an individual’s authorization. This policy also gives users rights to examine and obtain copies of their health records, request corrections, and appoint a covered entity to share their PHI to a third party [8].

In Europe, the GDPR is a data protection law applicable to organizations globally so long as they target or collect data related to individuals in the EU. This law is guided by seven protection and accountability principles focused on (1) lawfulness, fairness, and transparency; (2) purpose limitation; (3) data minimization; (4) accuracy; (5) storage limitation; (6) integrity and confidentiality; and (7) accountability through GDPR compliance [7]. GDPR likewise shares strict rules surrounding what constitutes a valid legal basis for sharing health data, including consent from a data subject.

However, this new era of digital technologies in healthcare may require updated data privacy laws to navigate the unique challenges posed by AI/ML-based SaMD, including the magnitude of training datasets often requiring large, diverse patient populations, the model interpretability challenges under both policies’ transparency requirements, and the maintenance of data subject rights such as deletion when data becomes embedded in trained models. There is also an ongoing discussion about whether traditional data consent frameworks can adequately cover entities such as complex AI systems, depending on a device’s use context and risk level, and how to incorporate existing principles of patient privacy throughout every stage of the product’s lifecycle.

## Medicolegal considerations of AI-assisted surgical practice

### Informed consent in the era of AI-assisted surgery

The ethical and legal doctrine of informed consent is predicated on the patient’s autonomous right to make decisions about their own body based on a thorough understanding of a proposed treatment. This requires the physician to disclose the nature of the procedure, its potential benefits and risks, and the reasonable alternatives. The introduction of AI-assisted surgery complicates nearly every aspect of this process.

As general AI literacy among the population is relatively low [9], patients may harbor preconceived notions shaped by the media, science fiction, and social media, leading to either unrealistic expectations of a futuristic, error-free procedure (i.e., “enchanted determinism” [9, 10]) or deep-seated fears of AI error. Thus, for medical care in which AI systems will be utilized to facilitate decision-making, operative planning, or to accomplish physical tasks, the surgeon must be sufficiently knowledgeable about the AI tool to explain it in understandable terms to the patient. Furthermore, surgeons should be equipped to have a discussion on reasonable alternatives to not using AI assistance in a patient’s care, offering data on how care might be different or whether there are any expected differences in outcome due to not using an AI system. This may become particularly important in the future should AI assistance become the dominant paradigm in healthcare.

Depending on the type and nature of the AI system used to facilitate care, an ethical informed consent discussion should likely encompass the unique risks introduced by an AI system itself [11]. This includes the risk of algorithmic bias leading to a suboptimal recommendation for that specific patient, the potential for technical malfunction or error, and the risks to data privacy and security inherent in using a data-driven system. As AI is incorporated into physical devices with the potential to perform autonomous functions, such as visual guidance, navigation, surgical assistance, or even discrete operative tasks, patients must understand the division of labor and responsibility in the operating room. The surgeon should clearly describe what role the AI plays – is it a diagnostic aid, a navigational guide, or a tool that actively participates in the procedure? The discussion should also cover the safeguards in place, such as the surgeon’s role in cross-checking and overriding the AI’s recommendations [12]. Similarly, surgeons should also disclose their training, qualifications, and experience with any AI systems to be used in the patient’s care [13].

### Liability and malpractice

Medical malpractice is a legal cause of action based on professional negligence, where a clinician’s actions deviate from the accepted "standard of care," resulting in patient harm. The standard of care is generally defined as the level of skill and care that a reasonably competent practitioner in the same specialty would provide under similar circumstances. The introduction of AI assistance into a patient’s care pathway complicates the determination of this standard and the attribution of fault when harm occurs.

One of the central issues relating to liability is decision ownership. In the current practice of surgery, the surgeon clearly owns any decisions made while delivering care. When an AI system provides a diagnostic assessment or a therapeutic recommendation upon which the surgeon acts, one must consider whether there are additional parties to whom responsibility could be assigned. If the AI’s recommendation was flawed and led to injury, was the surgeon negligent for following it? Would the surgeon have been negligent for ignoring a potentially superior recommendation? This further raises questions around culpability in medical decisions that involve AI assistance and result in patient injury [14]. We dive more deeply into considerations of liability allocation and culpability further below.

## Tripartite framework of AI risks in surgery

In developing a conceptual framework through which to understand risk associated with use of AI systems in surgery, we first note that risks do not necessarily arise from a single point of failure but rather from a complex interplay between the technology itself, the human user, and the environment in which it is deployed. The Massachusetts Institute of Technology AI Risk Repository offers a framework for which to consider domains of risk related to AI systems [15]. We categorize these risk domains into three distinct but interconnected categories that are more readily aligned with the use and deployment of AI systems in surgery: (A) inherent risks sourced from the AI system itself; (B) risks introduced by the clinician-user and other human factors; and (C) systemic risks arising from the institutional deployment process.

### Risks inherent to the use of AI systems

Risks introduced into clinical environments by AI systems themselves can be further categorized into risks related to data used and generated by the system, and risks introduced by the algorithm. Risks can be introduced via the algorithm’s training data as the data can have embedded biases that lead to poor performance on underrepresented patient populations. Poor training data quality (e.g., absent or incomplete data, inaccuracies, or annotation errors) can also negatively affect the recommendations of an AI system [16]. This can ultimately impact surgical decision-making, especially in cases where it is not feasible for the surgeon to evaluate the representativeness or quality of the training data on their own. AI systems that use Retrieval-Augmented Generation (RAG) to incorporate data from external sources introduce the risk of retrieval inconsistency or contamination: the model might pull outdated guidelines, irrelevant studies, or fail to retrieve the most pertinent information [17].

Inherent algorithmic risks include non-interpretability, where the reasoning behind an AI system’s recommendation is opaque, thereby making it difficult for the user to gauge if the model’s output should be incorporated into their judgement or not. Generative AI systems have a unique risk of hallucination—reporting of fabricated information—and confabulation—reporting plausible but incorrect information to fill in gaps in data – which may be difficult for users to detect. AI systems may be optimized for performance metrics that may not be clinically relevant, potentially achieving high overall accuracy while failing in specific important cases [18]. For example, a computer vision model may have excellent reported pixel-level predictive performance but still fail at identifying specific pathologies on images. These models are also susceptible to evolving medical practices, populations, and diseases, meaning their performance may change over time.

### Surgeon-and user-sourced risks

The introduction of AI tools into surgical workflows does *not* eliminate human factors from the risk equation; it transforms them. The interaction between a surgeon’s decisions and the AI’s output creates a new set of risks that originate not necessarily from flawed code or data, but from the human interpretation of the system’s output.

Surgeons, like all human experts, rely on a combination of rigorous training and cognitive heuristics, or mental shortcuts, to make decisions efficiently under pressure. However, these same heuristics can result in cognitive biases that lead to errors in perception or judgment. Madani et al. have described a conceptual framework for understanding intraoperative behaviors and performance that comprises five domains: psychomotor skills, declarative knowledge, advanced cognitive skills, interpersonal skills, and personal resourcefulness [19]. AI systems may influence or modify surgeon behaviors across these domains, and surgeons’ own perception of the ability and utility of these tools can result in risks.

The use of an AI decision support system creates a new dynamic where human biases can interact with the technology in potentially dangerous ways. Automation bias, the tendency for humans to over-rely on automated systems, can lead to surgeons deferring to the system’s output without sufficient critical scrutiny, passively accepting an incorrect recommendation from an AI tool and effectively abdicating their own expert judgment. This is particularly dangerous when the AI output is plausible but wrong. Furthermore, an AI system can amplify confirmation bias. A surgeon who has already formed a preliminary diagnosis may selectively pay attention to AI outputs that support their conclusion while downplaying or ignoring contradictory evidence. In this scenario, the AI’s potential to act as a "second opinion" and correct human error is completely negated as the surgeon is not using the AI to challenge their thinking, but to reinforce it. This is the modern version of Way et al.’s 2003 report that surgeons’ own visual perceptual heuristics during laparoscopic cholecystectomy were leading to bile duct injuries [20]. These surgeon-AI interaction risks are further accentuated by recent evidence suggesting that *how* an AI system presents its output can change a user’s trust and confidence in an AI system’s recommendations and thus the likelihood of a user performing additional steps to verify an AI system’s output [21].

The consequence of these interactive biases is a failure of the human-AI team. As currently conceptualized, surgical AI systems are designed with the assumption that the surgeon will provide critical oversight, yet cognitive biases can short-circuit this very function, leading to the uncritical acceptance of a flawed AI suggestion or the incorrect dismissal of a valid one, both of which can lead to patient harm [22].

The rise of large language models and vision-language models facilitates AI use as users no longer need programming knowledge to instruct or interact with AI systems. It is critical to recognize that general purpose commercial systems have not been evaluated or authorized by regulators such as the FDA as a clinical decision support system. Therefore, the outputs of these systems in the context of clinical decision support are unreliable and potentially dangerous [23]. Despite controls that developers place on these systems to prevent inappropriate clinical use, prompting techniques can circumvent these protections.

### Deployment sourced risks

A perfectly designed AI model used by a well-trained surgeon can still result in patient harm if it is deployed within a clinical environment that lacks the necessary governance, oversight, and safety infrastructure. These are systemic risks that are the responsibility of the healthcare organization in which it is being deployed. Given the relative novelty of AI assistance in surgical care, it is important to recognize that not all risks will be predictable. Risks from AI deployment are inevitable but can be mitigated through a robust, centralized organizational governance framework that recognizes the potential sources of risk that are introduced by the use of AI.

Traditionally, acquisition of new technologies for clinical care has been delegated to individual departments that are best equipped to recognize the changing needs of their physicians and the rapid evolution of clinical care within their own specialties or via multispecialty committees (e.g., perioperative procurement committee). Similarly, decisions about AI procurement and use may be made in silos, preventing a clear, organization-wide view of the associated risks and creating blind spots in safety oversight.

Institutions deploying AI systems must consider that AI systems may make mistakes. As previously discussed, these mistakes may be the result of inaccurate or inappropriate training data or choices made regarding the selection of models or the performance metrics of those models. Importantly, these mistakes (particularly those made by large language models) may be made with high confidence by an AI system and with less regularity or predictability than the types of errors otherwise made by humans. Institutions must carefully vet AI systems not only for their rate of errors but also for the type and nature of errors they may make [24]. Furthermore, outside of clinical use and risk of medical harm, it should be noted that the use of AI systems introduces the risk of violations of patient privacy and institutional harm through data breaches or system breaches that result in unwanted or unsafe outputs or system behaviors, as detailed further below.

## Clinical impacts of AI risks

The technical, human-factor, and systemic risks detailed in the previous section are not abstract concerns. When these risks materialize clinically, they translate into tangible, and often severe, consequences for patients, the integrity of the patient-physician relationship, and the quality of care. Table 1 offers a succinct summary of the clinical risks that may arise from AI systems.

**Table 1:** Types of risks that arise from use of AI systems and examples of how such risks may manifest in clinical application

Risks	Source	Definition	Surgical example
Training bias	Model	The AI model learns and amplifies existing disparities present in its training data, leading to inequitable performance across different patient populations	An AI tool for predicting post-operative complications, trained primarily on data from a Caucasian population, underestimates the risk for an African American patient
Hallucination	Model	A generative AI model produces fluent, confident-sounding output that is factually incorrect, misleading, or entirely fabricated	An LLM summarizing a pre-operative workup invents a "history of cardiac arrhythmia" for a patient with a healthy heart
Inappropriate metrics	Model	The model’s performance is measured using technical metrics that do not correlate with clinically meaningful outcomes or safety in the real world	A computer vision AI boasts 98% accuracy in identifying surgical events but fails to detect bleeding obscured by surgical smoke
Data poisoning	Model	Malicious actors intentionally corrupt the model’s training data, causing the AI to learn incorrect patterns and produce systematically flawed outputs	An attacker injects mislabeled pathology images into a training set, causing the AI to consistently classify malignant tumors as benign
RAG contamination	Model	The external knowledge base used by a Retrieval-Augmented Generation (RAG) system is compromised with false information, which the AI then retrieves and presents as fact	An external database of surgical guidelines is altered, causing a RAG-based decision support tool to recommend the wrong medication for a condition
Non-interpretability	Model	The "black box" nature of a deep learning model makes its internal decision-making logic opaque and incomprehensible to human users	An AI recommends an unconventional and high-risk surgical approach, but the surgeon is unable to understand the rationale behind the recommendation
Purpose limitation	Model	A generalist AI model (like an LLM) is used for a specific clinical task for which it has not been adequately trained, validated, or constrained	A surgeon uses a general-purpose chatbot to ask for a specific chemotherapy dosage, and the LLM provides an incorrect answer based on outdated web data
Cognitive biases	User	Inherent mental shortcuts (e.g., automation bias, confirmation bias) lead to the uncritical acceptance of flawed AI outputs or rejection of correct ones	A surgeon, anchored on their initial anatomic assessment, dismisses a correct but contradictory finding from an AI tool and cuts the common bile duct (confirmation bias)
Misuse/Misinterpretation	User	Use of an AI tool on the wrong patient population, for an unvalidated task, or misinterpreting its output due to inadequate training of the user	A surgeon applies a risk score model validated for elective hernia repair to an emergency trauma case, leading to a grossly inaccurate risk assessment
Insufficient governance	Deployment	The healthcare institution lacks a centralized system for vetting, monitoring, and managing AI tools, and has no incident response plan	An AI tool’s performance degrades over time (model drift), but there is no audit system in place to detect this until an adverse event occurs

### Medical errors

The primary clinical impact of AI risk is the potential for medical error, leading to patient harm and deviations from the established therapeutic standard of care. A missed or incorrect diagnosis is a critical risk, particularly with AI systems designed for diagnostic support or visual interpretation. An AI system’s failure can originate from incorrect training data, inappropriate generalization of its training data, or inappropriate interpretation of the AI system’s output. Inaccurate visual interpretation by a computer vision algorithm—whether caused by technical limitations, poor image quality, or compromised visibility during an operation (such as a blurred or obscured surgical field)—can lead to failure in identifying a malignant lesion, an important anatomical structure, or an intraoperative adverse event. This may result in misdiagnosis or iatrogenic injury.

Flawed AI recommendations can lead to incorrect or suboptimal treatment. Biased (intentional or unintentional) data can result in AI outputs that systematically discriminate against certain populations who are under or misrepresented in the data, resulting in unequal outcomes or reduced benefits in clinical outcomes [15]. A generative AI hallucination could suggest an inappropriate drug dosage, while a biased risk-prediction model could lead a surgeon to offer a procedure that is too aggressive or not aggressive enough for a particular patient. Reliance on AI-driven therapeutic deviations will breach the standard of care if the surgeon acted unreasonably in following the AI recommendation. Furthermore, as discussed previously, the use of a non-interpretable "black box" AI system would make it difficult, if not impossible, for a surgeon to provide a patient with a complete understanding of the rationale behind a recommended treatment. An adverse event that occurs following a procedure where consent was based on an opaque AI recommendation could be legally challenged as having been inadequately consented, creating both ethical and legal liability.

### Negative impact on patient outcomes

Even when a procedure is performed correctly, patients can still experience harm due to the unavoidable risks associated with the procedure. When using AI systems, new or different unavoidable risks might arise during treatment. If these risks occur and were properly explained to the patient beforehand, they would not automatically result in medical liability, even if the patient were to experience a complication. Nonetheless, these risks need to be carefully considered and mitigated. For example, a negative outcome could be a lack of response to a medication suggested by an AI system. While it may not result in an injury to the patient, the lack of response is suboptimal. A more complicated issue is when reasonable steps to reduce these risks are not taken. This could be seen as negligence and lead to legal liability, which could fall on the manufacturer, the healthcare organization, or the individual surgeon.

### Physician–patient relationship and therapeutic autonomy

Outside of direct patient outcomes, there may be additional negative impacts on the quality of surgical care. The practice of surgery is built upon a foundation of trust between the patient and the surgeon with the relationship characterized by human judgment, empathy, and shared decision-making. The introduction of AI, particularly if implemented poorly, may erode this relationship and compromise the autonomy of both the patient and the surgeon. If patients perceive that decisions are made by an impersonal algorithm rather than their trusted surgeon, it may threaten the patient’s trust in the surgeon and thus the basis of the physician–patient relationship [25]. Furthermore, a lack of transparency or an AI error that causes harm can permanently damage trust in the clinician, the technology, and the healthcare system as a whole.

AI potentially poses a dual threat to therapeutic autonomy. For patients, a lack of understanding of AI recommendations undermines their ability to participate in their own care. For surgeons, over-reliance on AI can diminish and devalue their clinical judgment and experience, leading to a "deskilling" effect where they become passive implementers of algorithms rather than experienced clinicians who can offer surgical recommendations tailored to an individual patient. This erosion of personal and professional autonomy is a significant concern for both parties and may have significant unintended consequences.

### Patient confidentiality and privacy

Integration of AI in medical practice comes with inherent risks to patient confidentiality and privacy. Legal frameworks determine when patient consent is required for using health data in AI training. In the U.S., HIPAA does not restrict the disclosure of patient data after deidentification, leaving patient data at risk for reidentification by cross-referencing multiple data sets [26]. On the other hand, GDPR in Europe governs the processing of all patient data that could be reasonably re-identified [27]. Additionally, the GPDR requires a valid legal basis for processing health data and imposes stricter requirements for protecting personal data, regardless of the legal basis for processing. While patient consent may not be required or appropriate as the legal basis for the primary use of health data for medical treatment, when consent is the basis for secondary uses of patient data under the GPDR, it must be explicit. This gap underscores the need to carefully consider the necessity and feasibility of obtaining a patient’s consent before their data is repurposed for AI development –distinct from the consent for the use of AI in a patient’s individual medical treatment.

Beyond consent, preserving privacy is fundamentally linked to maintaining patient autonomy by ensuring transparency in the use of AI-based medical decisions. Transparency is two-fold: AI developers must disclose how they use patient data to train medical AI systems, and clinicians must inform patients when AI is used to generate diagnosis or treatment recommendations [26]. While most data used to train medical AI are obtained from electronic health records, other sources of training data could include purchasing records, income data, criminal records, and social media [28]. Third-party access to such data can cause harm far beyond medical privacy violation, impacting employment and credit decisions, insurance access and rates, as well as causing social stigma and psychological harm for certain AI predictions related to future medical conditions [26, 28].

HIPAA’s narrow scope of regulating data sharing among covered entities (limited to healthcare providers and insurers) remains its primary weakness regarding data privacy [29]. This enables non-covered technology companies to share individual health data, user-generated, or health-adjacent data for research or commercial use. De-identified data is susceptible to re-identification by cross-referencing other available datasets. While states can individually impose stricter data protection policies, the lack of a comprehensive federal data protection framework both impacts the development of AI health technologies and compromises individuals’ privacy rights [30].

In contrast, the GDPR in Europe provides more robust safeguards by applying its protections to all entities that handle personal data, including natural persons and business entities [31]. The GDPR also prevents the processing of data for “automated individual decision making” that can have significant legal or personal consequences on the data subject. It also requires impact assessments, including risk assessments and risk mitigation and data protection strategies, which also apply to new AI-based technology in clinical health settings. Although the GDPR grants EU citizens stronger data protection than HIPAA provides to Americans, the inherently cross-border nature of AI accentuates the need for harmonized international regulation to ensure the ethical and secure use of personal health data in AI development and deployment [30].

## Liability allocation

While formal legal precedent is scarce, qualitative studies of surgeons reveal a general acceptance that the ultimate responsibility for the patient remains with them as the "captain of the ship." However, there is also a recognition that the introduction of AI adds layers of complexity that the current legal framework is likely not yet fully equipped to handle [32]. AI developers, healthcare systems, and clinicians are all stakeholders to whom liability may be allocated when an AI system is used in surgery.

Surgical AI developers can bear liability if their technology is defectively designed, inadequately tested, or fails to perform as advertised. In cases of patient injury, courts could hold the manufacturer liable for a flawed algorithm—for example, if coding errors or biased training data lead to incorrect recommendations. A developer that negligently designs or implements an AI system (e.g., failing to rigorously validate the model on appropriate test data) could be sued for negligence, since a poorly validated or unsafe algorithm endangers patients [33]. Beyond design defects, failure-to-warn is another avenue of liability. Surgical AI developers must provide clear instructions, warnings, and limitations for their products. If a surgical AI has known constraints (e.g., it is unvalidated for certain patient populations or scenarios), the developer must explicitly warn end-users. A manufacturer could be liable if a doctor was not informed that the AI has a limited scope or might not work under certain conditions.

Healthcare systems that deploy AI systems assume a share of responsibility for their safe integration. Institutions must vet the AI technology before adoption, ensure it is appropriate for their patient population, and, as previously noted, continue to monitor its performance. If a hospital uses an unverified or unreliable AI system, or one that has not been approved for the specific clinical context, the hospital could be found negligent. Furthermore, failing to maintain the AI system, apply necessary software updates or patches, or de-implement a system that is performing poorly could represent a breach of the institution’s duty to provide safe equipment. It is also important to note that companies may use contracts to allocate risk via disclaimers of liability and indemnity clauses when supplying AI systems to hospitals [33].

The operating surgeon remains the final decision-maker in the operating room, even with AI assistance. The introduction of AI does not absolve a surgeon of the duty to meet the standard of care. As emphasized earlier, if a surgeon relies on a flawed AI recommendation that a reasonably prudent surgeon would have recognized as incorrect, they can be found negligent for “blindly” following the AI. Courts have historically been reluctant to absolve physicians by blaming a tool or system; doctors have been held liable when relying on erroneous clinical guidelines or diagnostic software, with judges noting that the physician must independently verify critical information [34]. Interestingly, prior work that surveyed the general public on various scenarios of clinical AI use suggested that clinicians who reject an AI’s recommendation to deviate from the standard of care are not safer from assignment of liability in case of an adverse outcome [35]. Thus, surgeons cannot and should not abdicate their judgment to an AI system as the surgeon maintains a duty to meet the standard of care for the patient that they are treating in a given scenario.

**Fig. 1:**
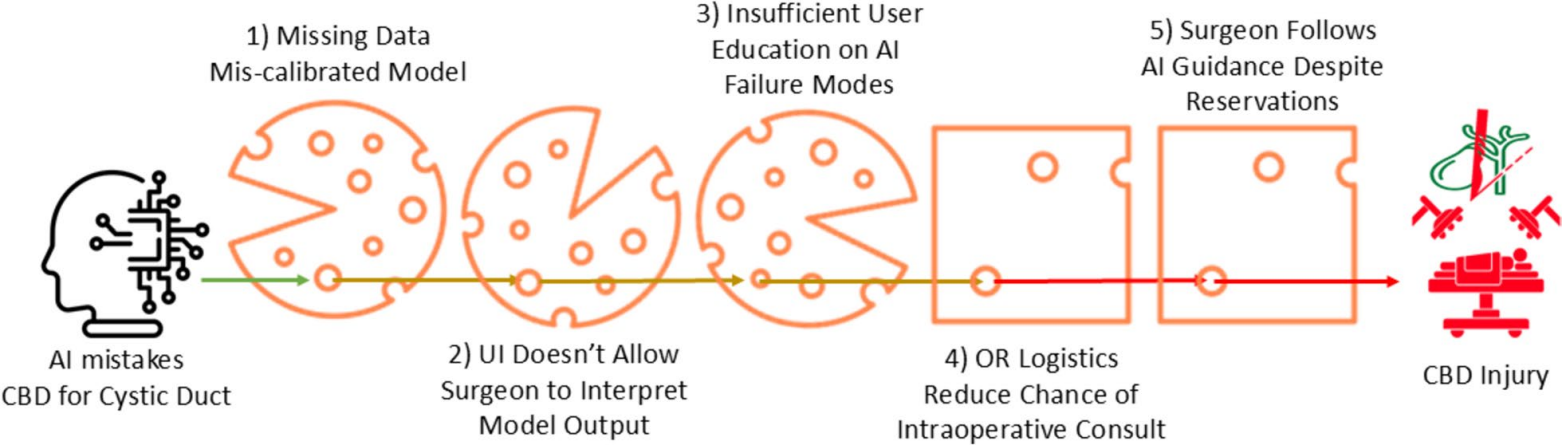
Swiss cheese model of an adverse event arising from a combination of developer (1,2), institutional (3, 4), and user (5) based factors that together reflect a systemic failure in preventing harm (e.g., common bile duct injury) from reaching a patient

With good governance practices and appropriate education and credentialing around the use of surgical AI systems, AI system failures should be amenable to mitigation by designing safe organizational systems to detect and prevent errors. The “Swiss cheese” model of accident causation has become ubiquitous in quality improvement. For AI models designed with human-in-the-loop safety checks (even for mostly autonomous systems), one can envision a scenario similar to that in Fig. 1, where multiple layers of failures at the AI development level, the institutional level, and the surgeon level align to result in an intraoperative adverse event. Taken together, these combined failures reflect systemic hazards and the potential for distributed liability in case of adverse events.

## Recommendations

To address the aforementioned risks associated with the implementation and utilization of AI systems, we recommend creating an AI Governance Blueprint to more clearly communicate and mitigate the potential harm to patients, clinicians, and institutions.

### Governance and incident response

At each institution, an appropriate governance committee of stakeholders, including AI and informatics experts, clinicians, and patients from various specialties, should be established to review AI systems. As institutions are responsible for training clinicians and staff on the proper use of equipment, the governance committee should ensure that AI developers have supplied comprehensive user manuals, training sessions, and safety guidelines with their products. Similar to how hospitals combine local credentialing with certification from specialty boards to assess a clinician’s clinical expertise, hospitals may look to specialty societies for guidance on appropriate training and credentialing in using specific AI tools. Institutions may consider partnering with specialty societies that have more specific expertise in the clinical applications and utilization of specific AI tools, such as those for surgical decision making or intraoperative guidance. Finally, implementation of appropriate risk-response systems is paramount [36]. Institutions should implement periodic performance audits to ensure no unexpected performance changes that could cause harm and would require de-implementing or re-training AI systems.

### Institutional AI formulary and oversight

Healthcare organizations should play a role in mitigating the risks associated with using AI systems in clinical practice by ensuring they have mechanisms for governance at both the ecosystem level and the point-of-care level. First, institutions should develop the expertise and systems necessary to govern AI models throughout their lifecycle. A key emerging role in this effort is the Chief Health AI Officer (CHAIO), who should be responsible for overseeing the ecosystem of clinical AI tools used within an institution [37]. This oversight includes vetting new AI models, serving as the operational home for existing ones, and conducting continuous surveillance of performance to ensure safety and effectiveness, which may involve phasing out underperforming models. At the point of care, healthcare organizations should build capacity to ensure that providers are using AI models safely and effectively in daily clinical practice. This mechanism could be comparable to how inpatient clinical pharmacists monitor the appropriate use of drugs in a hospital, ensuring that the technology is deployed correctly and its outputs are appropriately integrated into surgical decision-making [38]. While these measures may not solve the issue of liability when there is harm, they are important for decreasing the risk of clinical AI use in the first place.

### Credentialing and certification for specific model use

Unlike a new instrument that primarily involves psychomotor adaptation, proficiency with a surgical AI system requires a distinct set of cognitive competencies: understanding the model’s limitations, recognizing potential biases in its output, interpreting its data correctly, and knowing when to override its recommendations. For many deep learning models, the surgeon cannot fully interrogate the system’s reasoning. This opacity means that a surgeon’s "experience" is less about repeated practice and more about certified training on the system’s known failure modes, performance boundaries, and appropriate use cases.

There may be benefits to considering standardized credentialing and certification programs for clinicians using specific AI tools. By establishing a requirement for credentialing and certification to use specific AI tools for clinical care, it frames the duty of disclosure as an objective, binary confirmation of competence. Thus, in cases where harm to a patient occurs as a result of the use of an AI system, the failure to obtain and disclose a lack of formal certification for an AI tool used in a procedure could then be considered a breach of the informed consent doctrine, regardless of whether the ultimate patient harm resulted from a deviation in the procedural standard of care. This approach not only empowers patients with truly material information but also provides a clear, defensible benchmark of proficiency for surgeons and healthcare institutions alike, mitigating liability for stakeholders and fostering responsible innovation.

In addition to physician credentialing, tools themselves should be designed such that they are only allowed for use in appropriate clinical scenarios. For example, it is conceivable that a certified clinician might use a tool trained on patients with Crohn’s disease on a patient with ulcerative colitis (a closely related but distinct condition) and receive inaccurate output. Developers of AI systems meant for clinical use (including health IT vendors, in-house informatics teams, and academic researchers) should be required to build clear guardrails into their products that specify what types of clinical scenarios their model should and should not be used in to preempt cases where user misapplies the algorithm to the wrong type of patient, thereby de-risking the use of these tools for developers, users, health systems, and patients.

### Partnership with professional and specialty societies

As noted by the Brookings Institution, many AI tools may be outside of the scope of regulatory agencies such as the FDA, falling into an “oversight gap” that should be addressed to protect patients, clinicians, and institutions from inappropriate or dangerous implementation and utilization of AI tools. While some large health systems may have the resources to create bespoke oversight frameworks, practices, and tools, many hospitals and practices are likely under-resourced, both financially and technically, to take this on themselves. As such, partnership between health systems, private and group practices, specialty societies, and insurers will likely be necessary to optimize local, regional, and national oversight of AI implementation [39].

Organizations such as the Society of American Gastrointestinal and Endoscopic Surgeons have already worked to release recommendations and best practices around data capture [40], data annotation [41], data structure [42], and the concept of digital surgery more broadly [43]. Multidisciplinary consortia of clinicians, scientists, and engineers from academia and industry have also outlined consensus positions on the barriers to clinical implementation of surgical AI and data science [44, 45] and the risks associated with inappropriate utilization of metrics to assess the performance of AI models [46]. There is likely also a role in leveraging pre-existing, society-led, multi-institutional clinical databases facilitate post-market data sharing across institutions and industry to assess for generalizability, effectiveness, and model drift. Such collaborations will be critical in ensuring that the implementation and evaluation of surgical AI systems are safe, effective, and appropriate.

## Conclusion

The implementation of surgical AI systems is inherently associated with risks. As with all elements of clinical practice, risks are unavoidable but should be mitigated to reduce the risk of patient harm, ensure patient autonomy, and center beneficence. While there are many unanswered questions regarding assignment of liability should harm arise from the utilization of surgical AI systems, there are steps that regulators, specialty societies, health systems, and clinicians can take now to design implementation strategies with the goal of minimizing the risk of harm.

## Declarations

### Artificial intelligence tools

Artificial intelligence tools, including ChatGPT 5 Pro (OpenAI), Gemini 2.5 Pro (Google), and Grammarly (Grammarly), were utilized to assist with literature search, word choice, grammar, and spelling. The authors take responsibility for all content in this publication.
